# Impact of Soybean Bioactive Peptides on Growth, Lipid Metabolism, Antioxidant Ability, Molecular Responses, and Gut Microbiota of Oriental River Prawn (*Macrobrachium nipponense*) Fed with a Low-Fishmeal Diet

**DOI:** 10.3390/biology14010011

**Published:** 2024-12-26

**Authors:** Chang Yang, Bo Liu, Liangkun Pan, Dong Xia, Cunxin Sun, Xiaochuan Zheng, Peng Chen, He Hu, Qunlan Zhou

**Affiliations:** 1 Wuxi Fisheries College, Nanjing Agricultural University, Wuxi 214081, China; 2021113023@stu.njau.edu.cn (C.Y.); liub@ffrc.cn (B.L.); suncunxin@ffrc.cn (C.S.); 2 Key Laboratory of Aquatic Animal Nutrition and Health, Freshwater Fisheries Research Center, Chinese Academy of Fishery Science, Wuxi 214081, China; panlk@ffrc.cn (L.P.); xiad@ffrc.cn (D.X.); zhengxiaochuan@ffrc.cn (X.Z.); 3 Jiangsu FIELD Technology Co., Ltd., Huaian 223001, China; 19951989996@163.com (P.C.); huhewangyi001@163.com (H.H.)

**Keywords:** plant-derived peptide, fishmeal replacement, biomass increase, microbiome, gut-hepatopancreas axis

## Abstract

The substitution of fishmeal with high-level soybean meal in the diet of crustaceans usually induces lipid accumulation and oxidative stress in the hepatopancreas. Therefore, it is essential to alleviate these adverse effects. In the present study, SBPs were used to alleviate the negative effects of a fishmeal decrease on the growth performance, lipid metabolism, antioxidant capacity, and gut microbiota of oriental river prawn (*Macrobrachium nipponense*) in an 8-week feeding trial. Three isonitrogenic and isolipidic diets were prepared as follows: R (reference diet with 32% fishmeal), CT (control diet with 22% fishmeal), and SBP (22% fishmeal with 1.25 g/kg soybean bioactive peptides). The prawns (initial biomass per tank 17 g) were randomly divided into three groups with four replicates. The results showed that the low-fishmeal diet induced the following: (1) the inhibition of growth performance and survival of prawns; (2) an increase in triglyceride content in the hepatopancreas and hemolymph and downregulation of *carnitine palmitoyl transferase 1* (*cpt1*) gene expression; (3) a reduction in antioxidant enzymes’ activities and their genes expression levels and an increase malondialdehyde (MDA) content; and (4) an increase in the abundance of the conditional pathogen *Pseudomonas* in the gut. SBPs supplementation in the CT diet effectively alleviated most of the above adverse effects. SBPs enhanced inducible nitric oxide synthase (iNOS) activity to synthesize nitric oxide (NO) by activating the *imd*-*relish* pathway. Most importantly, SBPs increased the potential probiotic *Rikenellaceae_RC9_gut_group* abundance and decreased the abundance of the conditional pathogen *Pseudomonas* in the gut. In conclusion, SBPs supplementation can improve low-fishmeal-diet-induced growth inhibition by regulating the gut microbiota composition to ameliorate lipid deposition and oxidative stress and strengthen immune status in oriental river prawn.

## Simple Summary

Low-fishmeal diets cause lipid accumulation and oxidative stress, inhibiting growth in aquatic animals. Soybean bioactive peptides (SBPs) have shown great potential in improving growth, antioxidant capacity, and immunomodulation. Therefore, this study investigated the effects of dietary SBPs on the growth, lipid metabolism, antioxidant status, immune response, and gut microbiota of oriental river prawn fed with a low-fishmeal diet. The results showed that SBPs could alleviate growth inhibition and lipid oxidative stress by affecting microbiota in the gut, such as increasing the potential probiotic *Rikenellaceae_RC9_gut_group* abundance and decreasing the abundance of the conditional pathogen *Pseudomonas*.

## 1. Introduction

Fishmeal is always the major protein source in aquatic feed due to its balanced amino acid composition, high digestibility, and palatability. A shortage of fishmeal supply is emerging as the global aquaculture industry continues to expand [1]. Therefore, finding a high-quality and affordable protein source to replace fishmeal is becoming an inevitable necessity. Soybean meal has shown promising potential as a fishmeal replacement because of its reasonable amino acid composition, easy availability, and economical price [2]. However, over-substitution of fishmeal with soybean meal generally leads to poor growth performance and unhealthy immune status due to the existence of anti-nutritional factors [3]. Adverse effects induced by soybean meal on antioxidant capacity, immune response, and gut microbiota composition have been widely reported in fish such as turbot (*Scophthalmus maximus*) [4], Atlantic salmon (*Salmo salar* L.) [5], gilthead sea bream (*Sparus aurata* L.) [6], rainbow trout (*Oncorhynchus mykiss*) [7], and northern snakehead (*Channa argus* Cantor, 1842) [3], etc. Poor growth performance was observed in Chinese mitten crab (*Eriocheir sinensis*) [8], swimming crab (*Portunus pelagicus*) [9], and oriental river prawn (*Macrobrachium nipponense*) [10] due to low-fishmeal diets. However, studies on the effects of low-fishmeal diets on antioxidant and immune functions, as well as the gut microbiota, in crustaceans are still lacking.

The gut microbiota, considered a virtual organ, plays an important role in host metabolism and physiological functions [11], such as digestion and immunomodulation [12,13]. Diet is the most prominent factor affecting gut microbiota homeostasis. Once the balance is destroyed, it will have a negative impact on the host’s health [14]. Previous reports have shown that gut microbiota dysbiosis exhibits a strong correlation with lipid metabolism disorder or oxidative stress in the liver, and the disorder can be prevented after restoring the balance [15], which suggests that the gut microbiota serves as a mediator in regulating lipid metabolism and oxidative stress [16]. Meanwhile, the microbiota can make an axis with a number of extraintestinal organs. Among them, the theory of the “gut liver axis” has been widely assessed since it was put forward [17]. The metabolism and immune regulatory pathways in the liver can be well explained based on the symbiotic relationship between the gut microbiota and the liver [18]. In crustaceans, the hepatopancreas is an important location in regulating metabolism and immune responses [19]. Therefore, it is necessary to focus on the “gut microbiota hepatopancreas axis” in studies of crustaceans.

Peptides derived from plant protein sources, generally possessing 2 to 20 amino acids and a molecular weight < 6000 Da [20], have immunomodulatory and antioxidant biological activities due to their amino acid composition, sequence, molecular weight, and hydrophobicity [21]. Soybeans can be chosen as a preferable source to obtain bioactive peptides because of their favorable price and high protein content. Some reports have pointed out that soybean bioactive peptides (SBPs) exhibit positive effects on growth and digestive performance [22], antioxidant capacity [23], and immunomodulation [21] in both in vivo and in vitro experiments in mammals and fish. All the results showed that SBPs have unlimited possibilities as a diet additive for aquatic animals. However, more studies are needed to provide evidence and data support.

Oriental river prawn (*Macrobrachium nipponense*) is a popularly farmed species in China, known for its rich nutritional value and short maturation cycles, which is also sensitive to the ratio of fishmeal to soybean meal in its diet based on our previous study [10]. In this study, a fishmeal level of 22% is set as the low fishmeal threshold value since our previous study showed that the dietary fishmeal level of oriental river prawn should not be less than 25% [10,24]. A diet with 32% fishmeal (reference group, R), a diet with 22% fishmeal (control group, CT), and a CT diet supplemented with soybean bioactive peptides (SBPs) were used to evaluate the impact of SBPs on growth, lipid metabolism, antioxidant capacity, molecular responses, and gut microbiota of oriental river shrimp fed a low-fishmeal diet. Meanwhile, the correlations between the hepatopancreatic physiological and molecular parameters and gut microbiota were revealed to identify the possible mechanism. We hypothesized that supplementing SBPs in the low-fishmeal diet can promote lipid decomposition and enhance antioxidant and immune functions, as well as improve gut microbiota structure, in prawn. It will promote the growth performance of prawn to a certain extent and provide a reference for the SBPs application in aquaculture.

## 2. Materials and Methods

### 2.1. Study Site

The experiment was conducted in the indoor facility of Dapu breeding farm of the Freshwater Fisheries Research Center (31°19′35.35588″ N and 119°45′13.63901″ E), Chinese Academy of Fishery Sciences, Wuxi, China, for 8 weeks.

### 2.2. Diet Preparation and Proximate Composition Analysis

In this study, three isonitrogen (40% crude protein) and isolipid (8% crude lipid) diets, including the reference diet (R, with 32% fishmeal), control diet (CT, with 22% fishmeal), and soybean bioactive peptides’ diet (SBP, with 22% fishmeal supplied 0.125% SBPs), were designed using Microsoft Excel^®^. The composition and nutrient levels of experimental diets were shown in Table 1. The protein sources in the diets were fishmeal, soybean meal, rapeseed meal, peanut meal and blood meal, lipid sources were fish oil and soybean oil, and carbohydrate source was α-starch. SBP diet was formulated by supplying 1.25 g/kg SBPs based on CT diet, whose fishmeal level was reduced from 32% to 22% compared with the R diet. SBPs (Tian le tai, provided by Jiangsu FIELD Technology Co., Ltd., Huaian, China) was obtained by enzymolysis with soybean protease. The main components were shown in Table 2. Stachyose and raffinose in SBPs were detected by high-performance liquid chromatography (HPLC) method. The relative molecular weight distribution of peptides detected as Jiang et al. [25]. The small peptides with molecular weight 180–500 account about 32%.

After all ingredients were crushed and sieved through an 80-mesh, the powders of the raw materials were weighed and mixed thoroughly. Then, the mixture was mixed well with lipid sources and water (250 mL/kg diet) when the SBPs were added to the stiff dough with water. The wet pellets (1.0 mm) were produced by squeezing the dough through twin screw extruder (F-26, Guangzhou Huazhong Optical Mechanical and Electrical Technology Co., Ltd., Guangzhou, China). Finally, after 15 h air-drying at room temperature (about 30 °C), the diets were stored in a −20 °C refrigerator until use. The proximate composition of experimental diets was measured based on standard AOAC methods [26].

### 2.3. Feeding Trial and Experimental Conditions

All juvenile oriental river prawns were provided by the breeding farm of Freshwater Fisheries Research Center, Wuxi, China. After 14 days acclimation feeding with commercial diet (36% crude protein and 6% crude lipid), 720 healthy prawns with similar size were selected and randomly stocked in 12 tanks (100 cm height × 180 cm diameter) with 60 individuals per tank. The initial biomass of prawns per tank was about 17 g. Four replicates per diet were randomly distributed.

All prawns in each tank were fed three times daily (7:30, 12:30, and 17:00) for 8 weeks. The daily feeding amount is 3% of the biomass in tank. The residue was removed one hour after feeding and subsequently dried in an oven at 65 °C to calculate the feed intake. During the feeding trail, we exchanged about one-tenth of the total water volume in the tank every week. Water conditions were maintained as follows: water temperature: 25–28 °C, pH: 7.0–8.5, ammonia nitrogen: <0.2 mg/L, dissolved oxygen: ≥5.0 mg/L.

### 2.4. Sample Collection

At the end of the feeding trial, all prawns were fasted for 24 h. The number and weight of all prawns in each tank were measured. Six prawns per tank were randomly selected and put on the ice for anesthesia. The hemolymph was collected from the pericardial sinus using a 1 mL sterile syringe with anticoagulant (6.6 g trisodium citrate, 2.4 g citric acid, 7.35 g glucose and double-distilled water to 500 mL). The hemolymph and anticoagulant were mixed at a ratio of 1:1 (v:v) and centrifuged (4 °C 4000 rpm for 10 min) to collect the supernatant. Three pooled hemolymph samples were obtained per tank for biochemical analysis of lipid content determination. Hepatopancreas and gut samples were collected too. A total of six prawns per tank were randomly obtained to collect hepatopancreas samples. Three hepatopancreas samples were put into Eppendorf tubes and stored at −80 °C for further analyzing the lipid content, antioxidant enzymes, and immune parameters. Another three hepatopancreas samples were preserved in RNAios plus (Takara, Dalian, China) for relative gene expression detection. Six gut samples from the same tank were pooled as one sample (four pooled samples per treatment) for gut microbiota sequencing detection.

### 2.5. Growth Performance Calculations

Growth performance parameters were calculated according to Hasanthi and Lee [27], as follows:

Initial biomass (IBM, g/tank), total prawn weight in each tank before the feeding trial
Final biomass (FBM, g/tank), total prawn weight in each tank after the feeding trial
Biomass increase (BMI, g/tank) = Final biomass − Initial biomass
Final body weight (FBW, g/prawn) = Final biomass/Final number of prawns
Survival (SR, %) = 100 × (Final prawn number/Initial prawn number)
Feed conversion ratio (FCR) = Feed intake (g/tank)/Biomass increase

### 2.6. Biochemical ParametersAnalysis

#### 2.6.1. Lipid Contents in Hepatopancreas and Hemolymph Detection

The hepatopancreas (about 0.1 g) was homogenized in 0.9 mL of sterile 0.85% saline and then centrifuged at 4 °C, 3000 rpm for 10 min to obtain the supernatant. Total protein concentration was detected based on the Coomassie brilliant blue method by commercial kit (Nanjing Jiancheng Bioengineering Institute, Nanjing, China). Then, triglyceride (TG) and total cholesterol (TC) contents in the hepatopancreas were detected using kits from Nanjing Jiancheng Bioengineering Institute (Nanjing, China). The TG and TC contents in the hemolymph were detected by an automatic biochemical analyzer (Mindray BS-400, Shenzhen Mindray Bio-Medical Electronics Co., Ltd., Shenzhen, China) using the corresponding commercial kits.

#### 2.6.2. Antioxidant Capacity Detection

The activities of superoxide dismutase (SOD), glutathione peroxidase (GPX), catalase (CAT), and inducible nitric oxide synthase (iNOS), as well as the contents of malondialdehyde (MDA) and nitric oxide (NO), in the hepatopancreas were detected with kits from Nanjing Jiancheng Bioengineering Institute (Nangjing, China).

### 2.7. RT-PCR Detection

The total RNA extraction from the hepatopancreas sample, the extracted RNA’s quantity and quality estimation, and reverse transcription to cDNA were conducted as described in our previous study [28]. The cDNA was synthesized following the ExScript™ RT-PCR kit’s instruction (Dalian Takara Co., Ltd., Dalian, China). The cDNAs were amplified by PCR with TB Green^®^ Premix Ex Taq™ II (Tli RNaseH Plus) Kit using a CFX 96 Real-time PCR Detection System (Bio-rad, Hercules, CA, USA). The *β-actin* was used as house-keeping gene, and the sequences of all primers were synthesized by Shanghai Generay Biotech Co., Ltd. (Shanghai, China) and were listed in Table 3. The 2^−ΔΔCT^ method was used to quantify the relative gene expression.

### 2.8. Analysis of Gut Microbiota

#### 2.8.1. DNA Extraction and Illumina MiSeq

The E.Z.N.A.^®^ Soil DNA Kit (Omega Bio-Tek, Norcross, GA, USA) was used to directly extract total DNA of microbes from pooled gut samples. The V3-V4 region of microbial 16S rRNA gene was amplified by polymerase chain reaction (PCR) using universal primer 338 F (5′-ACTCCTACGGGAGGCAGCAG-3′) and 806 R (5′-GGAC TACHVGGGTWTCTAAT-3′). The purified library was built and sequenced using an Illumina MiSeq platform (Illumina, San Diego, CA, USA) after the PCR products were quantified, combined, and purified.

To obtain clean reads for further analysis, raw data after sequencing were merged by FLASH (version 1.2.11) [35]. And then, chimeric sequences were detected and removed to obtain high-quality clean tags using VSEARCH (version 2.13.4) [36]. UPARSE (version 7.1) was used to cluster high-quality sequences into amplicon sequence variants (ASVs) for bioinformatics analysis based on a 97% sequence similarity level. According to the Ribosomal Database Project (RDP) database [37], taxonomic classifications were annotated after selecting representative sequence of ASVs and screening each ASV.

#### 2.8.2. Richness, Diversity, and Composition Analysis of Gut Microbiota

Alpha-diversity indices (Observed species, Chao 1, Shannon), which reflected richness and diversity of gut microbial community in each sample, were analyzed using QIIME2 (version 2022.11). The Venn diagram and percent stacked column chart were conducted to display the shared and unique phyla or genera in each group and the species composition in each sample, respectively. The differences in species complexity among groups were assessed by principal coordinates analysis (PCoA) performed with R package (version 4.4.2).

#### 2.8.3. LEfSe Analysis of Gut Microbiota

The specific taxa microbes, i.e., biomarkers in different groups were identified by applying linear discrimination analysis effect size (LEfSe) using nonparametric Kruskal–Wallis and Wilcoxon rank-sum tests. The LDA score value threshold was set ≥3. Then, the differential analysis of gut microbes at the genus level was performed using STAMP (Statistical Analysis of Metagenomic Profiling) software (version 2.1.3). The same species of different gut microbes based on both analysis methods were found out and compared.

### 2.9. Evaluation Analysis

The parameters of lipid metabolism, antioxidant capacity, and dominant genera were submitted to do the Pearson’s correlation analysis. The *p*-value threshold was set <0.05. The heatmap created using R package (version 4.4.2) represented the correlation ship between microbiota at genus level and other parameters.

The genera whose abundances were in the top 50 in three groups were selected, and submitted to construct interspecies interaction network within group by Pearson’s correlation analysis. The absolute values of coefficient and *p*-value were ≥0.95 and <0.05 respectively. The visual networks were created using Gephi (version 0.9.7).

### 2.10. Statistical Analysis

Mean values of parameters of lipid contents, enzyme activities, and gene relative expressions of three samples in the same tank were submitted to SPSS (v.26.0) for statistical analysis. The results were shown as mean ± standard errors. All data were assessed using one-way analysis of variance (ANOVA) with Duncan’s adjustment firstly. Then, Student’s *t* test was used to assess the difference between two groups. Values with *p* < 0.05 were regarded as statistically significant.

## 3. Results

### 3.1. Growth Performance

**Figure 1:**
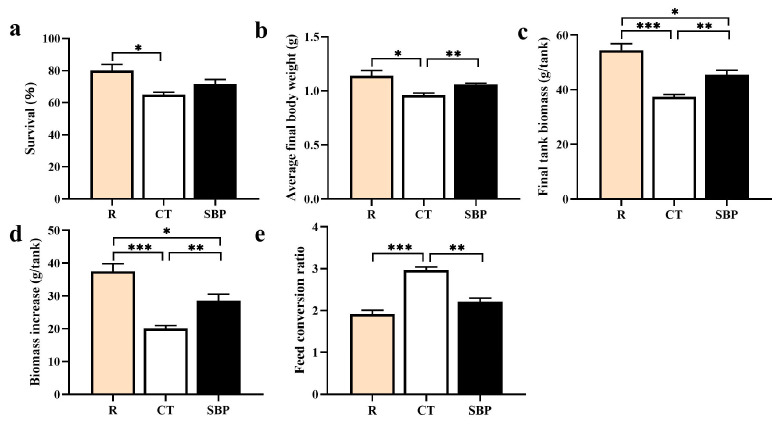
Effects of SBPs on growth performance of oriental river prawn: (**a**) survival; (**b**) average final body weight; (**c**) final biomass; (**d**) biomass increase; (**e**) feed conversion ratio. *n* = 4. * *p* < 0.05, ** *p* < 0.01, *** *p* < 0.001.

As shown in Figure 1, the SR of prawns in the CT group was significantly decreased than that in the R group (*p* < 0.05), while it increased more in the SBP group than in the CT group to a value similar to that in the R group without significant difference (*p* > 0.05). The FBW, FBM, and BMI of prawns in the CT group were significantly decreased compared to those in the R group (*p* < 0.001). The FBW, FBM, and BMI of prawns in the SBP group were significantly increased compared with those in the CT group (*p* < 0.01), though they were still significantly lower than those in the R group, except FBW (*p* < 0.01). Conversely, the FCR of prawns in the R and SBP group were significantly lower than that in the CT group (*p* < 0.01). There was no significant difference in FCR between the R and SBP groups (*p* > 0.05).

### 3.2. Biochemical Parameters

#### 3.2.1. Lipid Contents in Hepatopancreas and Hemolymph

**Figure 2:**
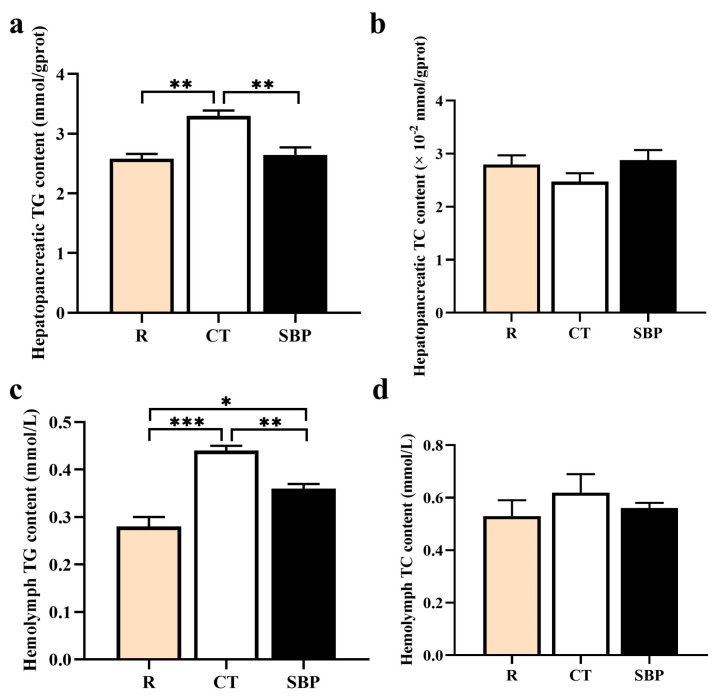
SBPs reduced lipid accumulation in the hepatopancreas of oriental river prawn fed with the low-fishmeal diet. (**a**–**d**) Assay of the hepatopancreatic and hemolymph triglyceride (TG) and total cholesterol (TC) (*n* = 4) (* *p* < 0.05, ** *p* < 0.01, *** *p* < 0.001).

The TG contents in the hepatopancreas and hemolymph of prawns fed with CT diet were significantly higher than that fed with R diet (*p* < 0.01), but TG content reduced significantly in the prawns supplemented with SBPs (*p* < 0.01) (Figure 2a,c), though the TG content in the hemolymph of prawns in SBP group was still significantly higher than that in the R group (*p* < 0.05). There was no difference on total cholesterol (TC) contents in the hepatopancreas and hemolymph among all groups (*p* > 0.05) (Figure 2b,d).

#### 3.2.2. Hepatopancreatic Antioxidative Capacity

**Figure 3:**
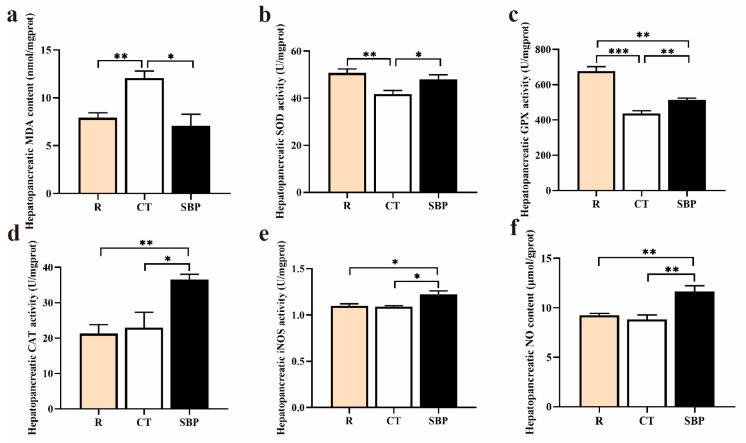
SBPs improved antioxidative capacity in prawns’ hepatopancreas: (**a**) malondialdehyde (MDA) content in the hepatopancreas (*n* = 4). (**b**–**d**) The hepatopancreatic superoxide dismutase (SOD), glutathione peroxidase (GPX), and catalase (CAT) activities (*n* = 4). (**e**,**f**) The inducible nitric oxide synthase (iNOS) activity and nitric oxide (NO) content in hepatopancreas (*n* = 4) (* *p* < 0.05, ** *p* < 0.01, *** *p* < 0.001).

The hepatopancreatic MDA content of prawns in the CT group was significantly higher than that in the R and SBP groups (*p* < 0.05) (Figure 3a). Prawns fed with the CT diet showed significantly lower SOD and GPX activities than those fed with the R diet (*p* < 0.05) (Figure 3b,c). Moreover, SOD and GPX activities were significantly increased in the prawns fed with SBP diet than those fed with CT diet (*p* < 0.05). However, the GPX activity of prawns in the SBP group was still lower than that in the R group (*p* < 0.05) (Figure 3d). SBPs supplementation in the diet significantly increased the CAT activity of prawns compared with those in the R and CT groups (*p* < 0.05), while no significant difference in CAT activity was found between prawns in R and CT groups (*p* > 0.05). Similar with CAT activity, SBPs supplementation increased the iNOS activity and NO content in the hepatopancreas than that in the R and CT groups (*p* < 0.05) (Figure 3e,f).

### 3.3. Relative Expression of Genes Related with Lipid Metabolism and Immunity

**Figure 4:**
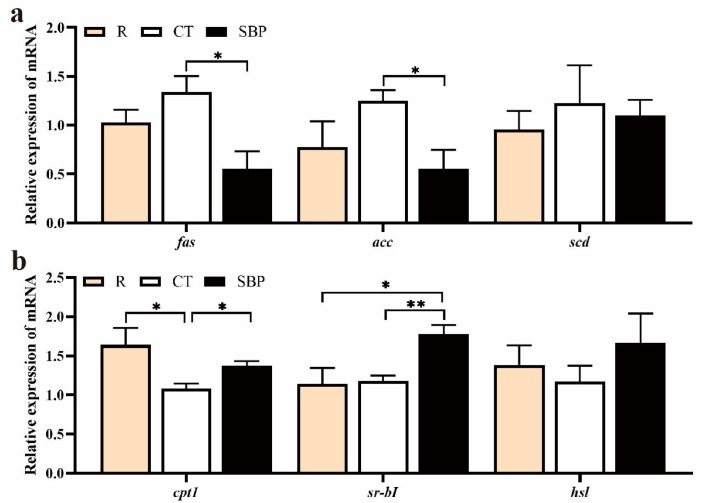
SBPs activated lipid catabolism genes’ expressions in hepatopancreas. (**a**) Relative expression of lipid synthesis genes in hepatopancreas (*n* = 4). *fas*, fatty acid synthase; *acc*, acetyl-CoA carboxylase; *scd*, acyl-CoA delta-9 desaturase; (**b**) Relative expression of lipid catabolism genes in hepatopancreas (*n* = 4) *cpt1*, carnitine palmitoyltransferase 1; *sr-b I*, scavenger receptor class B type I; *hsl*, hormone-sensitive triglayceride lipase. (* *p* < 0.05, ** *p* < 0.01).

For genes involved in lipid synthesis (Figure 4a), there were no significant differences in the relative expressions of *fas* and *acc* genes between R group and CT group, but their relative expressions were significantly downregulated in the SBP group compared with that in the CT group (*p* < 0.05). The mRNA level of *cpt1* (Figure 4b) was significantly inhibited in the prawns fed with CT diet compared with those fed with R diet. After supplementing SBPs in the CT diet, *cpt1* expression level was upregulated (*p* < 0.05). Moreover, SBPs supplementation in the diet significantly upregulated the *sr-b I* expression level more than that in the R and CT groups (*p* < 0.05).

**Figure 5:**
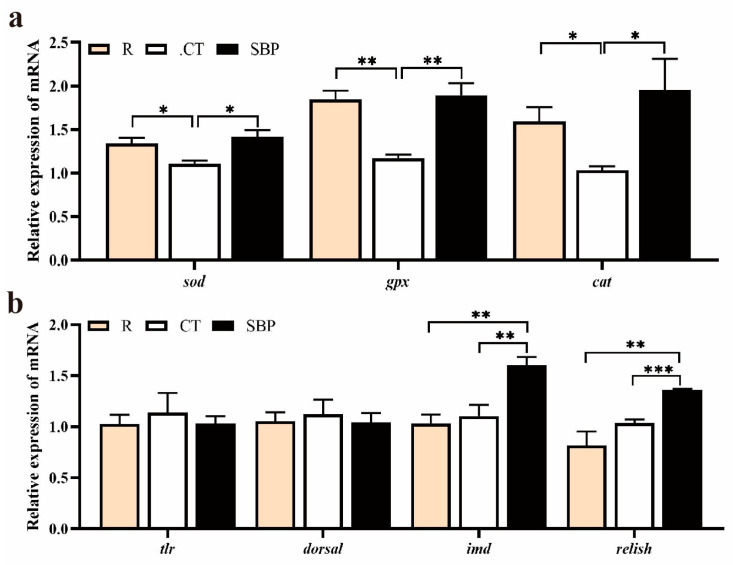
SBPs upregulated genes’ expressions on antioxidant enzymes and *imd*/*relish* in hepatopancreas. (**a**) Relative expression of genes on antioxidant enzymes in hepatopancreas (*n* = 4) (**b**) Relative expression of genes like *tlr*, *dorsal*, *imd*, and *relish* in hepatopancreas (*n* = 4) (* *p* < 0.05, ** *p* < 0.01, *** *p* < 0.001).

As shown in Figure 5a, the mRNA levels of antioxidant enzyme genes, like *sod*, *gpx*, and *cat*, were significantly inhibited in the CT group compared with those in the R and SBP groups (*p* < 0.05). No significant differences on the relative expression of *tlr* and *dorsal* were found in the hepatopancreas of prawns among all groups (*p* > 0.05). The mRNA levels of *imd* and *relish* in the hepatopancreas of prawns fed with SBP were significantly upregulated than those fed with the R and CT diets (*p* < 0.01) (Figure 5b).

### 3.4. Analysis of the Gut Microbiota

#### 3.4.1. Richness, Diversity, and Composition of the Gut Microbiota

**Figure 6:**
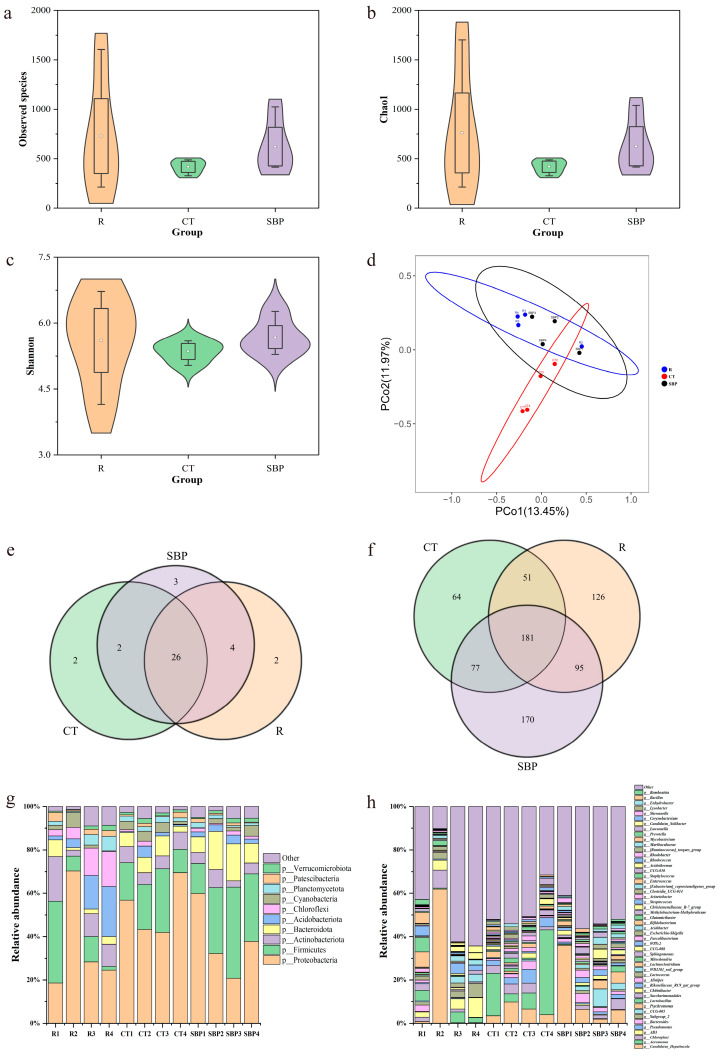
SBPs ameliorated the gut microbiota composition. (**a**–**c**) Observed species, Chao1 and Shannon index (*n* = 4). (**d**) Principal coordinates analysis between three groups. (**e**,**f**) Venn diagram comparing gut microbiota distribution at phylum and genus level. (**g**,**h**) Relative abundances of the major taxonomic lineages at phylum and genus level.

As presented in Figure 6a–c, there were no significant differences in the observed species, Chao 1 and Shannon indices of gut microbiota in the prawns among all groups. According to the PCoA plot (Figure 6d), there was a clear separation of gut microbiota communities between R and CT groups and the same between the CT and SBP groups. However, the gut microbiota in the SBP group had high similarities with that in the R group. According to the Venn diagrams (Figure 6e,f), compared the gut microbiota of prawns in the R and CT groups, 6 phyla and 221 genera were only observed in the R group, while 4 phyla and 141 genera were only found in the CT group. When the gut microbiota in the CT and SBP groups were compared, 2 distinct phyla and 115 distinct genera were only observed in the CT group, while 7 phyla and 260 genera only appeared in SBP group.

Proteobacteria, Firmicutes, Actinobacteriota, Bacteroidota, and Acidobacteriota were dominant phyla in the gut microbiota of oriental river prawn (Figure 6g,h). The most abundant phylum was Proteobacteria, with a relative abundance ranging from 35.43% ± 11.79% to 52.84% ± 6.48%. Compared with the microbiota in the CT group, the relative abundances of Firmicutes and Bacteroidota were increased in the SBP group. At genus level, *Candidatus_Hepatincola* (affiliated with Proteobacteria), *Aeromonas* (affiliated with Proteobacteria), *Chloroplast* (affiliated with Cyanobacteria), *AD3* (affiliated with Firmicutes), and *Pseudomonas* (affiliated with Proteobacteria) were the most abundant genera. Among them, the relative abundances of *Aeromonas* and *Pseudomonas* were increased in the CT group compared with those in the R group. After supplementing SBPs in the CT diet, their relative abundances reduced. *Rikenellaceae_RC9_gut_group* was a genus accounting a larger proportion in all treatments too. Its relative abundance was improved in the prawn fed with SBP diet compared with prawn fed with CT diet.

#### 3.4.2. LEfSe Analysis of Gut Microbiota to Identify Biomarker

**Figure 7:**
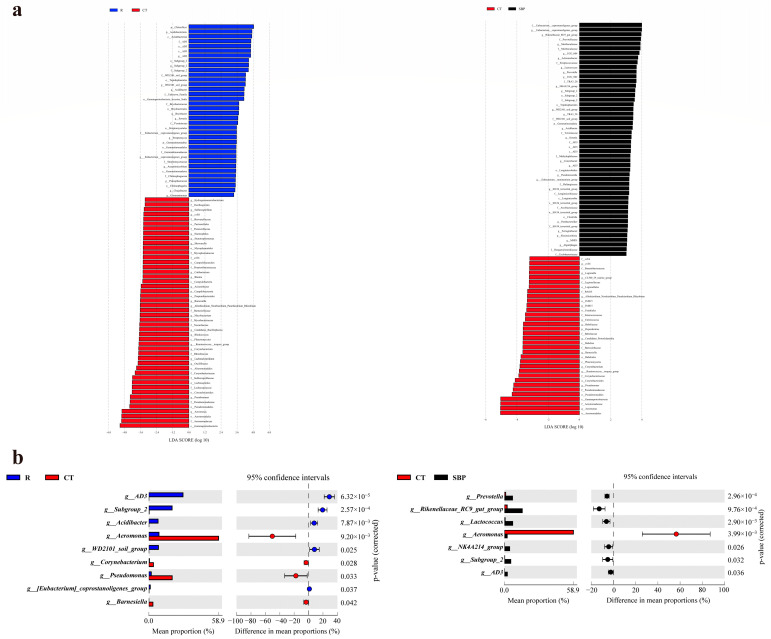
Analysis of microbiota differences between groups: (**a**) LEfSe analysis between R and CT as well as CT and SBP. Bar chart indicating the log-transformed LDA scores between groups, with a threshold of LDA > 3.0. (**b**) Bar diagram of altered microbes at genus level in the CT group compared with R group or SBP group; *p*-value < 0.05 represents a significant difference.

The LEfSe analysis was used to discover the significantly altered microbiota (Figure 7a). There were 36 distinct altered taxonomies in the R group, and 48 distinct altered taxonomies in the CT group when comparing the microbiota between the R and CT groups, while 34 and 50 distinct altered taxonomies were observed in the CT group and the SBP group, respectively, when comparing the microbiota between the CT group and the SBP group. Biomarkers in the CT group, including 2 classes (Planctomycetes, Gammaproteobacteria), 3 orders (Corynebacteriales, Pseudomonadales, Aeromonadales), 5 families (cvE6, Barnesiellaceae, Corynebacteriaceae, Pseudomonadaceae, Aeromonadaceae), and 6 genera (*cvE6*, *Corynebacterium*, *Barnesiella*, *Ruminococcus_torques_group*, *Pseudomonas*, *Aeromonas*), were altered when comparing the microbiota between the CT group and R, SBP groups.

Thereafter, a STAMP analysis was performed to identify and visualize genera with significant differences (Figure 7b). Compared with the R group, the abundances of *Aeromonas*, *Corynebacterium*, *Pseudomonas*, and *Barnesiella* were enriched significantly (*p* < 0.05) in the CT group, while the abundances of *AD3*, *Subgroup_2*, *Acidibacter*, *WD2101_soil_group*, *[Eubacterium]_coprostanoligenes_group* were significantly downregulated (*p* < 0.05). Compared with CT group, the abundance of *Aeromonas* was decreased significantly in the SBP group (*p* < 0.05), while the abundances of *Prevotella*, *Rikenellaceae_RC9_gut_group*, *Lactococcus*, *NK4A214_group*, and *Subgroup_2*, *AD3* were increased significantly (*p <* 0.05).

### 3.5. Correlation Analysis

**Figure 8:**
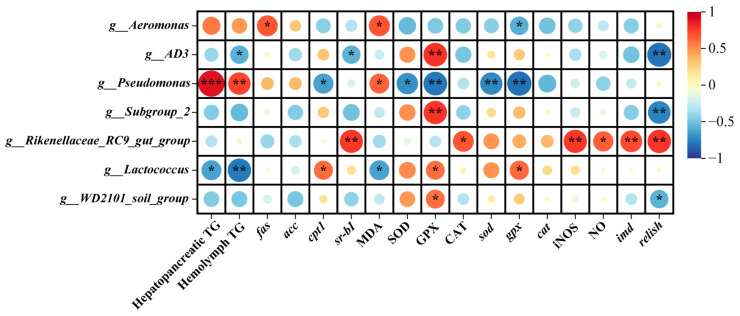
Correlation analyses between genera of gut microbiota and biochemical and molecular indexes. Red or green circles suggest positive or negative relations, respectively. Asterisks within the different circles represent significance, * *p* < 0.05, ** *p* < 0.01, *** *p* < 0.001.

The correlations between gut microbiota and lipid metabolism, antioxidant capacity and immune related gene relative expressions were explored (Figure 8). The MDA content and the relative expression level of *fas* showed positive correlations with the abundance of *Aeromonas* (*p* < 0.05); however, the relative expression level of *gpx* was negatively related with *Aeromonas* abundance (*p* < 0.05). The abundances of *Subgroup_2*, *AD3*, and *WD2101_soil_group* were positively related with the GPX activity (*p* < 0.05), while they were negatively related with the expression level of *relish* (*p* < 0.05). The *AD3* abundance exhibited negative relationship with hemolymph TG content and the expression level of *sr-b I* (*p* < 0.05). For *Rikenellaceae_RC9_gut_group*, its abundance increase upregulated the expression levels of *sr-b I*, *imd* and *relish*, CAT and iNOS activities, and NO content in the hepatopancreas (*p* < 0.05). The *Lactococcus* abundance was positively related with the activities of GPX and the expression levels of *cpt1* and *gpx* (*p* < 0.05)*,* whereas it was negatively related with the hepatopancreatic TG and MDA contents and hemolymph TG content (*p* < 0.05). Interestingly, the correlations between the *Pseudomonas* abundance and the above parameters were contrary to *Lactococcus* abundance. In addition, the abundance of *Pseudomonas* showed negative relationship with SOD activity and the expression level of *sod*. And *WD2101_soil_group* showed positive relation with the GPX activity and negative relation with the expression level of *relish*.

**Figure 9:**
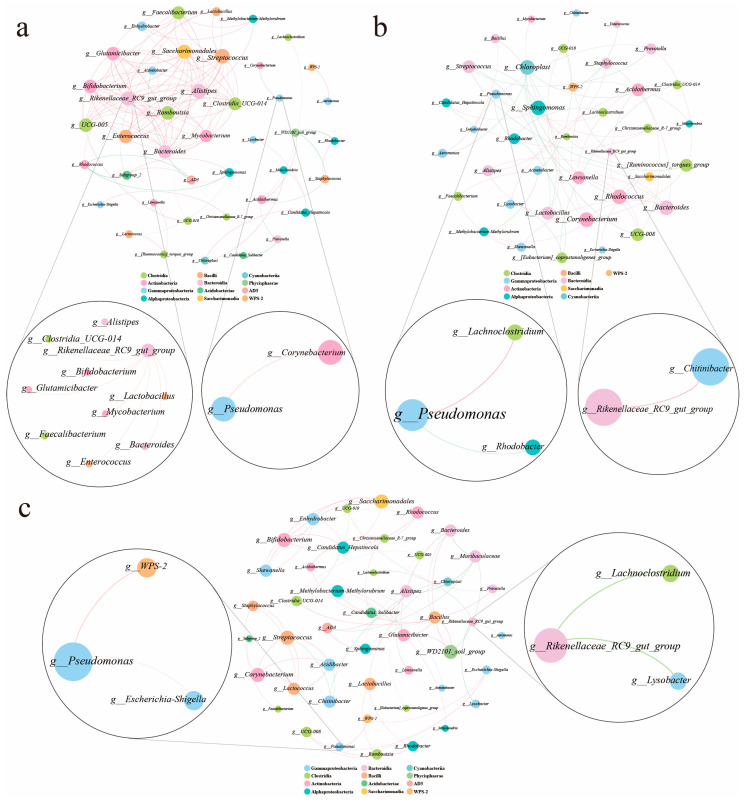
Ecological interaction network analysis of gut microbiota at genus level. (**a**–**c**) Interspecies interaction network of gut microbiota for oriental river prawn fed with different diet. Each node corresponds to a genus. Node colors represent genus belonged to different classes. A red or green edge suggests positive or negative correlations between two individual nodes.

From the correlations presented above, *Pseudomonas* and *Rikenellaceae_RC9_gut_group* were two very important genera that influenced the lipid metabolism and immunity. Therefore, *Pseudomonas* and *Rikenellaceae_RC9_gut_group* were mainly focused to analyze the correlation network with other microbiota to understand the interaction of microbiota at genera level. In the R group (Figure 9a), there were 113 links (edges) in total. Among them, *Rikenellaceae_RC9_gut_group* abundance was positively correlated with the other 9 genera abundances. There was a positive relation between *Pseudomonas* and *Corynebacterium* abundances. In the CT group (Figure 9b), a total of 51 links (edges) were observed. *Rikenellaceae_RC9_gut_group* abundance was just positively related with *Chitinibacter* abundance. *Pseudomonas* abundance was positively related with *Lachnoclostridium* abundance, while it was negatively related with *Rhodobacter* abundance. In the SBP group (Figure 9c), there were 68 links (edges). The abundance of *Pseudomonas* exhibited positive relations with the abundances of *WPS-2* and *Escherichia-Shigella*. Interestingly, *Rikenellaceae_RC9_gut_group* abundance was negatively related with *Lachnoclostridium* and *Lysobacter* abundances.

## 4. Discussion

### 4.1. SBPs Improved Growth Performance

SBPs exhibited potential benefits in mammals, including antioxidant [38] and immunomodulatory [39]. Undoubtedly, the improvements on antioxidant and immune functions were beneficial to the growth of animals. In this study, the effects on growth of SBPs were explored in oriental river prawn fed with a low fishmeal diet firstly. Previous study showed that the growth performance of prawn could be adversely affected when the fishmeal level in the diet was less than 25% due to soybean meal substitution [10], which was supported in this study. However, after supplying SBPs (1.25 g/kg diet) to the CT diet, the growth and feed utilization of prawn were increased. It might be because the SBPs may trigger the digestive enzyme activities, facilitating the digestion of feed nutrients, as demonstrated in study of Chinese mitten crab [25]. This study suggested that SBPs could improve the growth not only as fishmeal substitute but also as diet supplementation [40]. Host growth may be affected by a diverse range of factors, including metabolism, antioxidant, and immune status, etc. Therefore, this study investigated the relevant indicators on metabolism and antioxidant functions further.

### 4.2. Biochemical Parameters in Hepatopancreas and Hemolymph

#### 4.2.1. SBPs Reduced Lipid Accumulation in Hepatopancreas and Hemolymph

It is well known that deposition of protein and lipid is the main reason for growth. Therefore, some parameters on lipid metabolism in the hepatopancreas of oriental river prawn were evaluated. Nutritional metabolism of animals can be determined by hemolymph biochemical parameters [41]. The triglyceride contents in the liver and plasma of fish were decreased because of suppression of soybean product on lipogenic enzyme activities [42]. However, the triglyceride contents of the hepatopancreas and hemolymph were increased in this study when soybean meal replaced fishmeal at high levels. A similar result was found in silvery-black porgy [2]. This may be attributed to the unbalanced amino acid profiles of soybean meal, especially the low level of taurine and glycine, which participated in the synthesis of bile acids to decrease triglyceride [43]. With the SBPs supplementation, TG contents in the hepatopancreas and hemolymph were decreased. The reason may be that the higher digestibility of SBPs may stimulate the digestive enzymes. Some SBPs had been proven to inhibit pancreatic lipase activity [40]. Moreover, SBPs were found to have the ability to inhibit the reabsorption of bile acids in the digestive system to reduce the lipid level [44]. In this study, the abundance of *Pseudomonas* and *Lactococcus* was found to be positively and negatively related with the TG contents in the hepatopancreas and hemolymph, respectively, indicating that the change in gut microbiota composition was one of the factors contributing to the TG variation.

#### 4.2.2. SBPs Improved Antioxidant Capacity

Lipid accumulation in the hepatopancreas may cause oxidative damage, which weakens the antioxidant and immune systems [45]. This can be proven to some extent by the fact that prawns in the CT group obtained the lowest survival rate among the three groups. Then, some antioxidant activities in the hepatopancreas were determined in this study. The MDA content, as a biomarker of lipid peroxidation, was increased significantly due to the fishmeal decrease in the diet. This finding was similar with the studies of white shrimp (*Litopenaeus vannamei*) [46] and turbot [4]. The antioxidant enzymes’ activities, such as SOD and GPX, in the CT group were inhibited to some extent. This may be due to the overconsumption of antioxidant enzymes to prevent the oxidation caused by anti-nutritional factors in soybean meal [4]. And it might also relate to the selenium status in the diet [46]. With the addition of SBPs, the activities of SOD, GPX, and CAT were improved. Some plant-protein-derived peptides could activate the antioxidant system and prevent lipid peroxidation to decrease the peroxidation products, such as MDA [47]. It was proved that amino acids, such as histidine, tryptophan, phenylalanine, proline, glycine, lysine, isoleucine, and valine, possess strong scavenging activity of free radicals; however, peptides are more effective as antioxidants than amino acids because they play a better role in free radical scavenging, metal complexing, and aldehyde-adducting activity [47]. Studies have also proven in animals that peptides from plant sources, including soybean, could reduce the MDA content and increase the antioxidant enzymes’ (SOD, CAT, GPX) activities [48]. Particularly for invertebrates without an adaptive immune system, the innate immune system is essential for survival [49]. There are some substances that could activate the immune system in plant-derived peptides. Taking the components of SBPs in this study as an example, stachyose [50] and raffinose [51] had been proven to exhibit the effect of enhancing animal immune systems. Most importantly, several peptides derived from soybean had immunomodulatory activity [21]. There were no significant differences in the immunologic factors assessed in this study between the R and CT groups. This may be due to the fact that the level of soybean meal instead of fishmeal did not exceed the tolerance of prawn’s immune system. Nevertheless, the iNOS activity and NO content were increased significantly after SBPs supplementation. A previous study revealed that this phenomenon was to prevent the immune system from overreacting [21].

### 4.3. Relative Expression of Genes Related Lipid Metabolism and Immunity

The mechanism of lipid metabolism at a molecular level was investigated in this study, which is closely related to the antioxidant and immune status in vivo [45]. The fatty acid synthesis genes, such as *fas* and *acc*, in the hepatopancreas would be upregulated due to the low fishmeal in the diet according to the previous report on oriental river prawn [10]. Their expression levels were increased in the CT group than the R group in this study too, but without significant difference. Some of SBPs, such as KNPQLR, EITPEKNPQLR, and RKQEEDEDEEQQRE, had been proven to be the fatty acid synthase inhibitory peptides [52]. Therefore, the expression levels of *fas* and *acc* in the prawns’ hepatopancreas were significantly downregulated due to the SBPs supplementation in this study. Moreover, as a key factor in lipid catabolism, *cpt1* was significantly inhibited in the CT group compared with the R and SBP groups. This may explain the significant reduction in TG content in hepatopancreas and hemolymph in the R and SBP groups. From the relationship heatmap, it could be clearly observed that the upregulation of *cpt1* and the downregulation of *fas* were partly due to the abundances of *Aeromonas* and *Pseudomonas* decrease as well as the abundance of *Lactococcus* increase. These results indicated that lipid metabolism in hepatopancreas was partly regulated by the gut microbiota.

The reasons of antioxidant enzymes and immune factors changes are revealed at the molecular level too. The genes expression levels of *sod* and *gpx* in the CT group were downregulated to some extent. With the addition of SBPs, the gene expressions of *sod*, *gpx*, and *cat* were activated. The changing trends on the expression level of these antioxidant enzyme’s genes were consistent with the variations in the corresponding enzymes’ activities too. The transcription factor NF-κB activation was a prerequisite for the increase in iNOS activity [32]. Thus, the expression levels of *imd* and *relish*, as family members in the NF-κB signaling pathway, were improved with the SBPs supplement in this study, partly leading to the iNOS activity and NO content increase. The *Rikenellaceae_RC9_gut_group* may participate in regulating *imd*-*relish* pathway based on the correlation analysis in this study.

### 4.4. SBPs Altered the Gut Microbiota

#### 4.4.1. SBPs Altered the Richness, Diversity, and Composition of the Gut Microbiota

The balance of the gut microbiota is closely related to animal health, which plays a crucial role in metabolism and immunity to maintain the homeostasis of the internal environment [53]. In this study, there was a clear difference in the gut microbiota community composition in CT group compared with the R and SBP groups based on the β-diversity analysis. It was proven by the interaction of the microbiota. Links were considered as a sign of complexity in the gut microbiota [54]. The minimum links in the CT group revealed a reduction in the complexity of the gut microbiota ecology. After supplementing SBPs, the number of links was increased. The complex network contributed to resisting the invasion of external strains [55]. It is necessary to find the key genera, which determine the complex of gut microbiota, in further study.

At the phylum level, the dominant phyla in all groups were Proteobacteria, Firmicutes, Actinobacteriota, Bacteroidota, and Acidobacteriota, respectively, which was similar with studies of white shrimp [56]. Among them, the highest abundance phylum was Proteobacteria, consistent with the result of Ding et al. [57]. The composition of gut microbial communities varies in response to diet, environmental conditions, and other factors [58]. In this study, the abundances of Firmicutes and Bacteroidota in the SBP group were higher than those in the CT group. These two phyla have been found to be involved in keeping gut immune homeostasis and enhancing animal health [59]. Therefore, results in this study supported that SBPs supplementation can be benefit gut health in prawns. Among the top 50 genera, the relative abundances of conditional pathogens, such as *Aeromonas* and *Pseudomonas* [60], were significantly higher in CT group than those in the other two groups. It was similar with the report that *Aeromonas* in the Atlantic salmon (*Salmo salar*) gut was increased, while soybean meal replaced fish meal in diet with a high level [61]. Moreover, Aeromonadaceae was found to be related to gut microbiota community dysbiosis [48].

#### 4.4.2. Biomarkers of the Gut Microbiota

Based on LEfSe analysis, the genera *Corynebacterium* and *Ruminococcus_torques_group* were the biomarkers in the prawn fed the CT diet, and they had been proven to be opportunistic pathogens [62] that break gut barrier integrity [63]. According to the results of STAMP analysis, the alter trend in *AD3* and *Subgroup_2* abundances was opposite to that of *Aeromonas*. It could be inferred that these two genera are potential probiotics combined with the health status of prawn. In addition, SBPs supplementation significantly increased the *Rikenellaceae_RC9_gut_group* and *Lactococcus* abundances in the gut. Noticeably, the *Rikenellaceae_RC9_gut_group* affiliated with the family Rikenellaceae was found to be positively related with acetate production [64]. Acetate can reduce lipid deposition by synthesizing uridine as the most abundant short-chain fatty acids in the gut [45]. *Rikenellaceae_RC9_gut_group* was proved to be related to lipid reduction [65]. There are few studies on the effects of *Lactococcus* on crustaceans. But it was reported as a safe microbe since they can enhance fish development and health [66].

### 4.5. Correlation Analysis of the Gut Microbiota

Two genera, *Pseudomonas* and *Rikenellaceae_RC9_gut_group*, are focused on in the correlation analysis. In the gut microbiota ecological networks, *Rikenellaceae_RC9_gut_group* showed positive correlations with the other nine genera in the R group. However, the complex of the network was weakened clearly in the CT group. At the same time, the network of *Pseudomonas* was enhanced with its abundance increasing in the CT group. After supplementing SBPs in the CT diet, the *Rikenellaceae_RC9_gut_group* showed negative correlations with the other two genera. It is worth noting that *Rikenellaceae_RC9_gut_group* exhibited negative correlation with *Lachnoclostridium* in the SBP group when *Pseudomonas* was positively correlated with *Lachnoclostridium* in the CT group. It was supposed that the decrease in the fishmeal in this study would disrupt the balance of the gut microbiota, and SBPs supply can increase the abundance of potential probiotic *Rikenellaceae_RC9_gut_group* to inhibit conditional pathogen *Pseudomonas* and restore the gut environmental homeostasis to a certain extent. According to the correlation analysis and the theory of “gut-liver axis” [67], the variation in gut microbiota ecological networks was an important factor affecting the metabolism and immunity in the hepatopancreas. But a modulatory mechanism is required for further study.

## 5. Conclusions

In summary, SBPs supplementation in a low-fishmeal diet can increase the abundance of potential probiotic *Rikenellaceae_RC9_gut_group* to decrease the conditional pathogen *Pseudomonas* abundance in the gut. The changes in these bacteria boosted antioxidant enzymes activities, weakened oxidative stress, and activated the *imd*-*relish* pathway to raise the immune status via the gut–hepatopancreas axis. This suggests that SBPs is an effective additive with benefit to prawn growth and health.

## Figures and Tables

**Table 1:** The composition and nutrient levels of experimental diets (%, dry matter).

Ingredients	R	CT	SBP
Fishmeal ^a^	32.00	22.00	22.00
Soybean meal ^a^	10.00	24.00	24.00
Rapeseed meal ^a^	10.00	10.00	10.00
Peanut meal ^a^	10.00	10.00	10.00
Blood meal ^a^	6.00	6.00	6.00
α-starch ^b^	23.20	18.70	18.575
Fish oil ^a^	2.00	2.25	2.25
Soybean oil ^a^	2.00	2.25	2.25
Monocalcium phosphate ^a^	2.00	2.00	2.00
Choline chloride ^c^	0.30	0.30	0.30
Vitamin and mineral premix ^c^	1.00	1.00	1.00
Bentonite ^c^	1.00	1.00	1.00
Salt	0.50	0.50	0.50
Soybean bioactive peptides ^d^	0.00	0.00	0.125
Proximate Composition			
Dry matter	91.53	92.32	92.43
Crude protein	39.75	39.84	39.91
Crude lipid	7.65	7.82	7.93
Ash	9.94	9.81	9.78
Fiber	2.65	3.30	3.31
Gross energy (MJ/kg)	18.50	18.70	18.75

Notes: ^a^ Provided by Jiangsu Fuyuda Food Products Co., Ltd., Yangzhou, China; ^b^ Provided by Foshan Guonong Starch Co., Ltd., Foshan, China; ^c^ Provided by Wuxi Hanove Animal Health Products Co., Ltd., Wuxi, China. ^d^ Provided by Jiangsu FIELD Technology Co., Ltd., Huaian, China.

**Table 2:** Main components and levels in soybean-derived bioactive peptides (SBPs).

Components (g/L)	Soybean-Derived Bioactive Peptides
Crude protein, ≥	40.0
Crude fiber, ≥	10.0
Crude lipid, ≥	15
Acid soluble protein (% of total protein), ≥	30
Stachyose, ≤	0.5
Raffinose, ≤	0.5
pH	3.5–4.5

**Table 3:** Real-time qPCR primer sequence.

Gene	Primer Sequence (5′→3′)	Accession Number /Reference
*β-actin*	F: GTGCCCATCTACGAGGGTTA	[29]
	R: CGTCAGGGAGCTCGTAAGAC	
*fas*	F: CGGTCAGACAAACTACGGCT	[10]
	R: CACTGAATAGCCACCCCAGG	
*acc*	F: CAAGGTCCACTACATGGTCT	[29]
	R: ACTCTTCCCAAACTCTCTCC	
*cpt1*	F: AATTTTTGACTGGCTTCTCC	[29]
	R: TCCATTCTGGAAATCATCTG	
*sr-b I*	F: TTATCCCTGGTGTGAATGTG	KP658863
	R: GAACTCTTCCCATTCCAACT	
*hsl*	F: GAAGGCCAGCGCTAATTTCG	MK633965.1
	R: TCGAACCACCCATGAGAAGC	[30]
*scd*	F: ATAATGTTTGCCCTGCTACA	KU922943.1
	R: ATGTCATTCTGGAAGGCAAT	[29]
*sod*	F: AGTTTCAGCCGTCTGTTCG	[31]
	R: CACAGTGCTTACATCACCCTTA	
*gpx*	F: CCTGGCTTTCCCCTGTAACC	[31]
	R: ACCGAGTCATCCGAAGGCA	
*cat*	F: GAACTGGGATTTGGTTGGCA	[31]
	R: GGTCCGAGAAAAGGATGGTG	
*tlr*	F: CGACCTCCACGACAACAAGA	[32]
	R: AAAGTTCCTGCACCAATGCG	
*dorsal*	F: TACGACCAACGGACAAGAGC	[33]
	R: CGCATTGTTGCTGTTTCCCA	
*imd*	F: GGCACCAAGCCTTCTTTTCAG	[34]
	R: ATATCCTTCGGGTCGCATTTC	
*relish*	F: TGCCAGACAGCCTTAAACGAT	[33]
	R: CTTGGAGGGTGGGTCATTGT	

Notes: *fas*, fatty acid synthase; *acc*, acetyl-CoA carboxylase; *cpt1*, carnitine palmitoyltransferase 1; *sr-b I*, scavenger receptor class B type I; *hsl*, hormone-sensitive triglyceride lipase; *scd*, acyl-CoA delta-9 desaturase; *sod*, superoxide dismutase; *gpx*, glutathione peroxidase; *cat*, catalase; *tlr*, Toll-like receptor; *imd*, immune deficiency.

## Data Availability

Data will be made available on request. The 16S rRNA sequencing data in this study have been uploaded URL: https://www.ncbi.nlm.nih.gov/bioproject/PRJNA1105913 (accessed on 29 April 2024) with accession number of PRJNA1105913.
